# Expression of Concern: microRNA-613 exerts anti-angiogenic effect on nasopharyngeal carcinoma cells through inactivating the AKT signaling pathway by downregulating FN1

**DOI:** 10.1042/BSR-20182196_EOC

**Published:** 2020-07-02

**Keywords:** AKT signaling pathway, FN1, Invasion, MicroRNA-613, Migration, Nasopharyngeal carcinoma

The Editorial Office has been made aware of potential issues surrounding the scientific validity of this paper, hence has issued an expression of concern to notify readers whilst the Editorial Office investigates.

